# mHealth-Based Just-in-Time Adaptive Intervention to Improve the Physical Activity Levels of Individuals With Spinal Cord Injury: Protocol for a Randomized Controlled Trial

**DOI:** 10.2196/57699

**Published:** 2024-06-28

**Authors:** Rachel L Carey, Ha Le, Donna L Coffman, Inbal Nahum-Shani, Mohanraj Thirumalai, Cole Hagen, Laura A Baehr, Mary Schmidt-Read, Marlyn S R Lamboy, Stephanie A Kolakowsky-Hayner, Ralph J Marino, Stephen S Intille, Shivayogi V Hiremath

**Affiliations:** 1 Department of Health and Rehabilitation Sciences Temple University Philadelphia, PA United States; 2 Khoury College of Computer Sciences Northeastern University Boston, MA United States; 3 Department of Psychology University of South Carolina Columbia, SC United States; 4 Institute for Social Research University of Michigan Ann Arbor, MI United States; 5 Division of Preventive Medicine, Heersink School of Medicine The University of Alabama at Birmingham Birmingham, AL United States; 6 Magee Rehabilitation Hospital Jefferson Health Philadelphia, PA United States; 7 MossRehab Hospital Jefferson Health Philadelphia, PA United States; 8 Good Shepherd Rehabilitation Network Allentown, PA United States; 9 Department of Rehabilitation Medicine Thomas Jefferson University Philadelphia, PA United States

**Keywords:** spinal cord injury, physical activity, just-in-time adaptive intervention, mobile health, randomized controlled trial, microrandomized trial, wearable sensors, ecological momentary assessment, community, mobile phone

## Abstract

**Background:**

The lack of regular physical activity (PA) in individuals with spinal cord injury (SCI) in the United States is an ongoing health crisis. Regular PA and exercise-based interventions have been linked with improved outcomes and healthier lifestyles among those with SCI. Providing people with an accurate estimate of their everyday PA level can promote PA. Furthermore, PA tracking can be combined with mobile health technology such as smartphones and smartwatches to provide a just-in-time adaptive intervention (JITAI) for individuals with SCI as they go about everyday life. A JITAI can prompt an individual to set a PA goal or provide feedback about their PA levels.

**Objective:**

The primary aim of this study is to investigate whether minutes of moderate-intensity PA among individuals with SCI can be increased by integrating a JITAI with a web-based PA intervention (WI) program. The WI program is a 14-week web-based PA program widely recommended for individuals with disabilities. A secondary aim is to investigate the benefit of a JITAI on proximal PA, defined as minutes of moderate-intensity PA within 120 minutes of a PA feedback prompt.

**Methods:**

Individuals with SCI (N=196) will be randomized to a WI arm or a WI+JITAI arm. Within the WI+JITAI arm, a microrandomized trial will be used to randomize participants several times a day to different tailored feedback and PA recommendations. Participants will take part in the 24-week study from their home environment in the community. The study has three phases: (1) baseline, (2) WI program with or without JITAI, and (3) PA sustainability. Participants will provide survey-based information at the initial meeting and at the end of weeks 2, 8, 16, and 24. Participants will be asked to wear a smartwatch every day for ≥12 hours for the duration of the study.

**Results:**

Recruitment and enrollment began in May 2023. Data analysis is expected to be completed within 6 months of finishing participant data collection.

**Conclusions:**

The JITAI has the potential to achieve long-term PA performance by delivering tailored, just-in-time feedback based on the person’s actual PA behavior rather than a generic PA recommendation. New insights from this study may guide intervention designers to develop engaging PA interventions for individuals with disability.

**Trial Registration:**

ClinicalTrials.gov NCT05317832; https://clinicaltrials.gov/study/NCT05317832

**International Registered Report Identifier (IRRID):**

DERR1-10.2196/57699

## Introduction

### Background

Physical inactivity is a significant concern among the >300,000 individuals living with spinal cord injury (SCI) in the United States [1], who are at an elevated risk of mortality due to cardiovascular diseases, diabetes, and lung disease [2-4]. Low levels of physical activity (PA) in individuals with SCI have also been associated with secondary health conditions such as pain, fatigue, and depression [5-7]. Research studies have found varying percentages of the population with SCI that perform regular PA [8-10]. On the higher end, Rauch et al [8] found that 48.9% of 485 participants with SCI in Switzerland met World Health Organization recommendations of 2.5 hours per week of moderate-intensity or higher PA, and 18.6% were physically inactive. On the lower end, Tasiemski et al [10] found that only 20% of 985 people with SCI in the United Kingdom performed regular PA of 2 hours per week, and 53.3% reported no regular PA. Furthermore, several other studies have indicated that most individuals with SCI do not perform regular PA [11-13].

Regular PA and exercise interventions in individuals with SCI have been linked with improved cardiorespiratory fitness, quality of life, and functional independence, as well as reduced risk of cardiometabolic disease, depression, and shoulder pain [11,12,14,15]. Although there are significant health and quality-of-life benefits with regular PA, various barriers limit individuals with SCI from performing PA regularly [16]. To address some of these barriers, the National Center on Health, Physical Activity, and Disability (NCHPAD) has developed a free web-based PA program for people with mobility limitations, chronic health conditions, and physical disabilities [17]. Web-based PA programs can lead to an increase in PA level and duration of PA performed by individuals with SCI [18-21]. One of the facilitators that can promote PA during PA intervention programs is to provide people with an accurate estimate of their everyday PA level in the community [18,22-24]. This information can empower individuals with knowledge about their regular PA levels, which in turn can help develop a physically active identity [16].

The PA of individuals with SCI is frequently assessed through samples of individuals who provide self-reports [8-10], which may have recall bias and social acceptability bias. To address these limitations and to better quantify PA and exercise interventions, researchers have validated sensor-based activity monitors in laboratories and community-dwelling environments [22,23,25]. PA tracking can also be combined with mobile health (mHealth) technology to provide a just-in-time adaptive intervention (JITAI) for individuals with SCI as they go about everyday life. A JITAI uses mHealth technology to deliver the intervention at appropriate times and contexts to support individuals’ health behaviors [24,26,27]. For example, Klasnja et al [27] used a JITAI to provide a real-time feedback intervention aimed at improving step count in individuals without disabilities. Our team tested a pilot JITAI for its feasibility and acceptability in individuals with SCI [24]. Results from our pilot study indicated that participants wished to have the system provide customized messages to help them further increase their PA levels. These customized messages combined with web-based PA intervention programs have the potential to impact factors such as intent to increase PA, awareness of PA benefits, reminders, and encouragement that are related to increased PA levels in individuals with SCI [11,12,18,28].

### Objectives

The overarching goal of this study is to investigate the benefits of combining an mHealth-based JITAI with the NCHPAD’s 14-week web-based PA intervention (WI) program for increasing and sustaining moderate-intensity or higher PA levels among individuals with SCI. This protocol outlines the study design for investigating the impact of integrating a JITAI with the WI program via a randomized controlled trial that integrates a 2-arm trial and a microrandomized trial (MRT). Specifically, individuals with SCI (N=196) will be randomized to either a WI program or a WI program combined with the JITAI (WI+JITAI). Within the WI+JITAI arm, an MRT [27,29,30] will be used to microrandomize participants several times a day to various types of feedback and PA recommendations. The primary aim is to investigate the long-term benefits of adding a JITAI to WI. We hypothesize that the integration of a JITAI into WI will produce significantly higher PA levels than those with WI alone. The secondary aim is to investigate the benefit of just-in-time PA feedback on proximal PA. Proximal PA is defined as moderate-intensity PA within 120 minutes of a PA feedback prompt. The exploratory aim will investigate time-invariant moderators such as biological sex and level of injury and time-varying moderators such as prior PA and engagement with the JITAI to help identify subgroups of individuals with SCI who are likely to benefit the most from the integration of a JITAI with WI.

## Methods

### Ethical Considerations

The institutional review board at Temple University has reviewed and approved this research study (protocol number 27338). The study will be executed in accordance with the ethical standards for human participants. All participants will provide written informed consent. The consent form will include the purpose of the research study, description of what participation in the study entails, and contact information of the principal investigator and the institutional review board. The consent form will be discussed with all participants by an investigator, and the participant will be offered a chance to consent at the conclusion of this discussion. Should the potential participant wish additional time to consider whether to consent, they will be given as much time as they desire. All participants will receive a copy of the consent form for their records. Verbal discussion will ensure participants understand each consent topic as it is discussed, and participants will be advised that their signature indicates their understanding and consent. It is reinforced throughout the consent form that participants are free to ask questions at any stage of the study, including before, during, or after the consent process. All participants will be informed of their right to withdraw from the study at any time. To maintain confidentiality, records and sensor data will be deidentified by assigning a case number to each participant. The study is registered on ClinicalTrials.gov (NCT05317832).

### Research Design

#### Overview

Participants will take part in the study from their home environment in the community. The study has three phases: (1) baseline (weeks 1 and 2); (2) WI program with or without JITAI (weeks 3-16); and (3) PA sustainability (weeks 17-24). Participants will provide survey-based information at various time points during the 24-week study. Participants will be asked to wear a smartwatch every day for ≥12 hours for the duration of the study. The PA intervention for this study is informed by the capability, opportunity, and motivation (COM-B) model, which highlights that these 3 constructs should be considered when developing a behavioral intervention [31]. To our knowledge, this is the first study to optimize a JITAI that has the potential to boost PA levels using real-time feedback for individuals with SCI.

#### Behavioral Intervention Model

**Figure 1:**
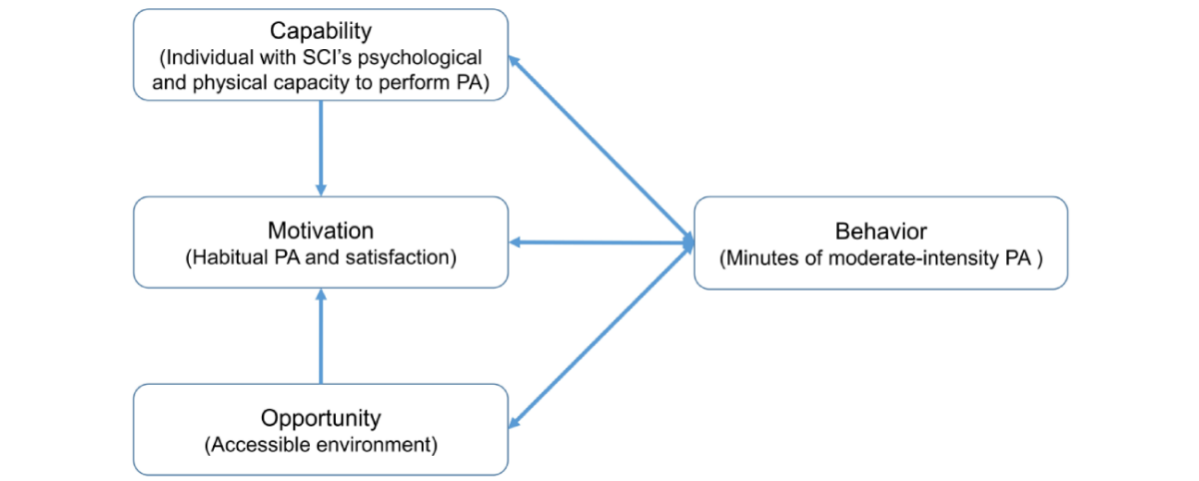
The proposed intervention builds on the capability, motivation, and opportunity model—a framework for understanding behavior (adapted from Michie et al [31], which is published under Creative Commons Attribution 2.0 International License [37]). The solid arrows represent potential influence between system components. An individual’s capability, motivation, and opportunity interact to generate behavior (eg, physical activity [PA]) that in turn influences these 3 constructs. SCI: spinal cord injury.

The proposed intervention approach builds on the COM-B model [31]—an organizing framework for designing behavior change interventions [32-34]. An individual’s capability, opportunity, and motivation interact to generate behavior (eg, PA) that in turn influences these 3 constructs (Figure 1) [31]. Capability is the individual’s psychological and physical capacity to perform the desired activity, including having the necessary knowledge and skills. Motivation is the individual’s internal drive or energy that directs behavior, including conscious or analytic decision-making, as well as habitual processes and emotional responses. Opportunity captures environmental (social and physical) factors external to the individual that facilitate or undermine behavior. The solid arrows in Figure 1 represent potential influence between these constructs [31]. For example, opportunity can enhance (eg, a supportive environment) or inhibit (eg, an adverse environment) motivation and capabilities [31]. It is also possible that enacting a behavior can alter capability, motivation, and opportunity. This framework incorporates context naturally through the “opportunity” construct [31]. For individuals with SCI, the context is characterized by physiological and environmental barriers to participate in PA [16,35] and stigma [36], which can hinder motivation and capability to engage in PA.

#### Conceptual Framework of the JITAI

**Figure 2:**
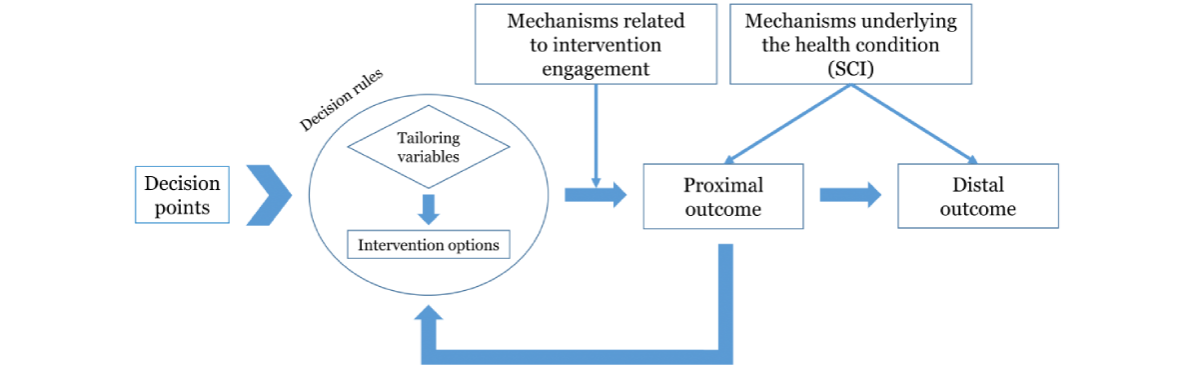
Conceptual framework of just-in-time adaptive intervention components including decision points, intervention options, tailoring variables, and decision rules for our proposed study (adapted from Nahum-Shani et al [26], which is published under Creative Commons Attribution Noncommercial International License [39]). SCI: spinal cord injury.

The framework used for this proposed study (Figure 2) is adapted from Nahum-Shani et al [26], which highlights 4 components including decision points, intervention options, tailoring variables, and decision rules. JITAIs often provide an intervention through a smartphone, wearable activity monitor, or both. The system automatically determines which intervention option, such as prompting an individual to set a goal to perform PA or prompting feedback about PA, could be helpful or especially effective. Engagement with the JITAI, that is, effort invested in the JITAI [38], is critical for intervention effectiveness [26]; therefore, JITAI components should be selected and designed to promote engagement [27].

#### NCHPAD’s WI Program

Our proposed JITAI will be offered along with NCHPAD’s 14 Weeks to a Healthier You Program [17,40,41], modified to be accessible on a smartphone or tablet. This 14-week WI program was launched in 2011 by NCHPAD with funding from the Centers for Disease Control and Prevention [40]. Since its launch, the program has been used by >45,000 people with various physical disabilities. It is the most widely recommended web-based PA program across various disabilities and state disability programs [42]. The program’s uniqueness lies in its ability to offer tailored exercise that matches the participants’ functional capabilities. The WI program was developed with guidance from literature and practice in health behavior change and internet-based health promotion programming. In addition, preliminary evaluations by individuals with disabilities were used to update the WI program. Textbox 1 lists motivational resource for each week of the program [41]. The content for all weeks include an introductory video, exercise resources, motivational resources, recipes, and nutrition resources. Customizable features for all weeks include my schedule, initial assessment, badge reward system, my goals, and daily reminder.

**Motivational resources for each week of the National Center on Health, Physical Activity, and Disability’s 14-week web-based physical activity program.**

**Motivational resources**

- Week 1: Goal setting
- Week 2: Overcoming barriers
- Week 3: Benefits of activity
- Week 4: Weight management
- Week 5: Self-monitoring
- Week 6: Keep it fun!
- Week 7: Rewarding yourself
- Week 8: Building social support
- Week 9: Stimulus control
- Week 10: Preventing injuries
- Week 11: Self-advocacy in recreation
- Week 12: Activity in daily life
- Week 13: Avoiding overtraining
- Week 14: Keeping it up!

#### Integration of JITAI With WI

The integration between the WI and JITAI is designed to blend multiple evidence-based components aimed at prompting capability, motivation, and opportunity for engaging in PA among individuals with SCI (Table 1). Capability will be enhanced by not only providing tailored information and advice for individuals with SCI via the standard WI but also by using self-regulatory techniques, operationalized by setting goals, monitoring progress, and providing timely feedback via the JITAI [24,26,31]. To enhance motivation to engage in PA, the JITAI will reinforce progress by providing just-in-time feedback about the minutes of moderate-intensity PA achieved or remaining to be achieved toward the daily goal. Opportunity will be enhanced by identifying specific types of goals (tailored, standard, or goal not presented) and PA feedback (achieved, to-go, or feedback not presented) that will be most beneficial in promoting daily and proximal PA, respectively. Although the standard goal encourages the individual to perform 20 minutes of moderate-intensity PA for the day based on PA guidelines for adults with SCI [43], a tailored goal is based on average minutes of moderate-intensity PA performed by the individual over the last 7 days. The choice of the last 7 days is based on PA guidelines for adults with SCI [43] and our pilot study [24] in which participants had lower PA for 1 or 2 days during the week. Although the achieved PA feedback message focuses on the PA level the person has accomplished until that moment in the day, the to-go message focuses on the remainder of the PA level the person has to accomplish to meet their designated goal. Prior research has indicated that the gain-framed messages were more likely than loss-framed messages to encourage prevention behaviors such as PA [44]. In this study, we are interested in evaluating whether participants prefer achieved PA message (gained) or to-go message (to gain) to accomplish their goal. In addition, the participants will be reminded to engage with the WI on a regular basis.

**Table 1:** Evidence-based components of the capability, opportunity, and motivation model targeted by the web-based physical activity intervention (WI) arm and WI+just-in-time adaptive intervention (JITAI) arm.

	WI	WI+JITAI
Capability	Tailored information and advice Weekly goal setting Monitoring weekly progress	WI: tailored information and advice, weekly goal setting, monitoring weekly progress JITAI: goal setting for the day, just-in-time tailored PA^a^ information
Motivation	Badges for completing weekly modules on WI PA progress over the last 7 days	WI: badges for completing weekly modules on WI; PA progress over the last 7 days JITAI: PA recommendations during goal setting; just-in-time tailored PA feedback messages; availability of real-time PA information; goal attainment at the end of day
Opportunity	Daily reminder to complete WI program	WI: daily reminder to complete WI program JITAI: just-in-time specific type of feedback to encourage proximal (within 120 min) PA achievement; just-in-time feedback to encourage more distal (daily goal) PA achievement

^a^PA: physical activity.

### PA Intervention Components

The PA intervention components include either the WI program or the WI program combined with JITAI for individuals assigned to the WI or WI+JITAI arms, respectively.

#### WI Program

**Figure 3:**
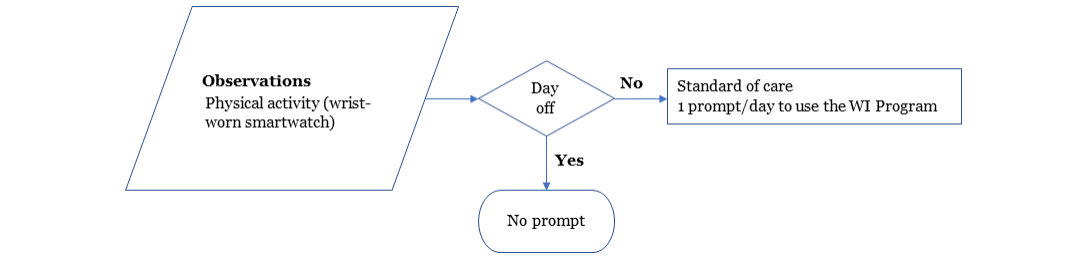
Schedule of reminders delivered over a week on a smartwatch. Participants will receive a daily reminder, except for the day off, on the smartwatch to access the web-based physical activity intervention (WI) program.

**Figure 4:**
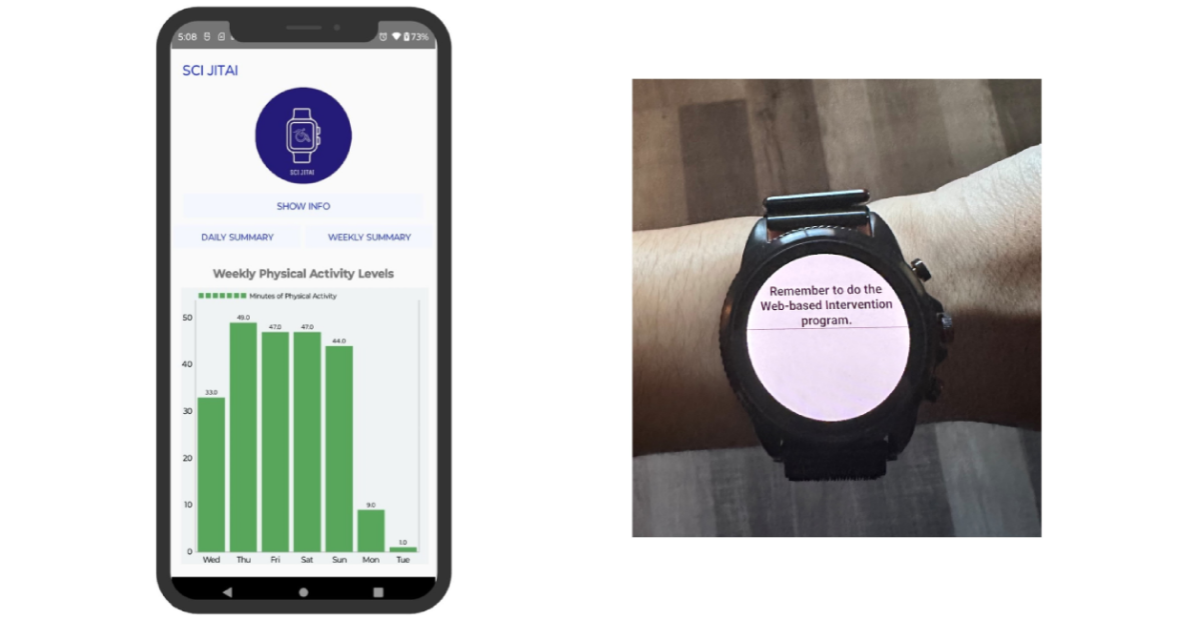
Example of feedback and reminders of physical activity. Left: participants will have access to physical activity levels performed over the last 7 days on the smartphone. Right: participants will receive a daily reminder, except for the day off, on the smartwatch to access the web-based physical activity intervention program.

Participants will access the 14-week WI program and answer questions related to their interests, needs, and abilities (Textbox 1), which will be used to tailor the content (exercises and videos). Participants can monitor the WI program use on their dashboard. From this point forward, program content automatically updates every week to introduce new exercises and a weekly motivational and educational topic. Participants will receive a daily reminder, except for the day off, on the smartwatch to access the WI program (Figure 3). Furthermore, participants will have access to the minutes of moderate-intensity PA performed over the last 7 days on the study smartphone (Figure 4).

#### JITAI Components

Participants in the WI+JITAI arm will have access to the JITAI components. In addition to WI program access, participants will receive just-in-time feedback and PA recommendations informed by the guidelines for adults with SCI [43]. Participants will have access to the minutes of moderate-intensity PA performed over the last 7 days on the study phone. In addition, they will have near–real-time access to minutes of moderate-intensity PA performed for the day on their smartwatch.

**Figure 5:**
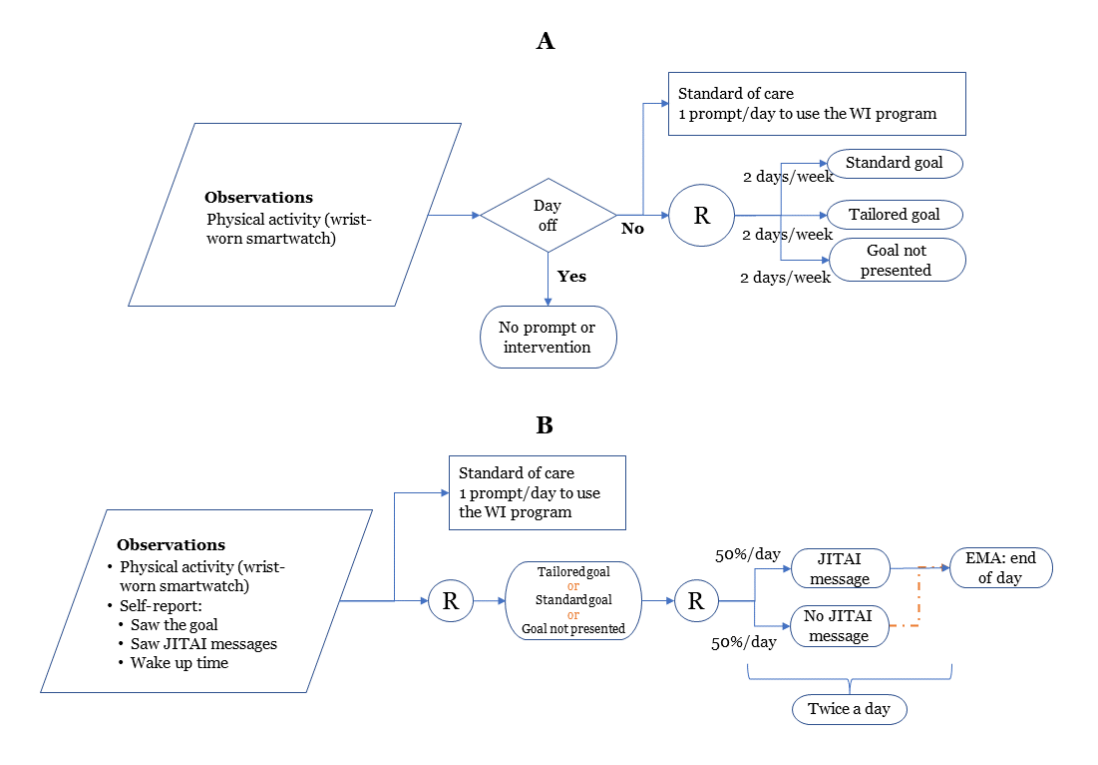
Schedule of reminders, just-in-time adaptive intervention (JITAI) messages, and physical activity recommendations delivered on a smartwatch. (A) Study flow over a week: participants will receive a daily reminder, except for the day off, to access the web-based physical activity intervention (WI) program. Participants will be randomized to receive a standard or tailored physical activity goal, or no goal, for 2 of the days each week. (B) Study flow over a day: participants in the intervention group. Participants will be randomized twice during each day to receive or not receive a JITAI feedback prompt. EMA: ecological momentary assessment; R: randomized.

**Figure 6:**
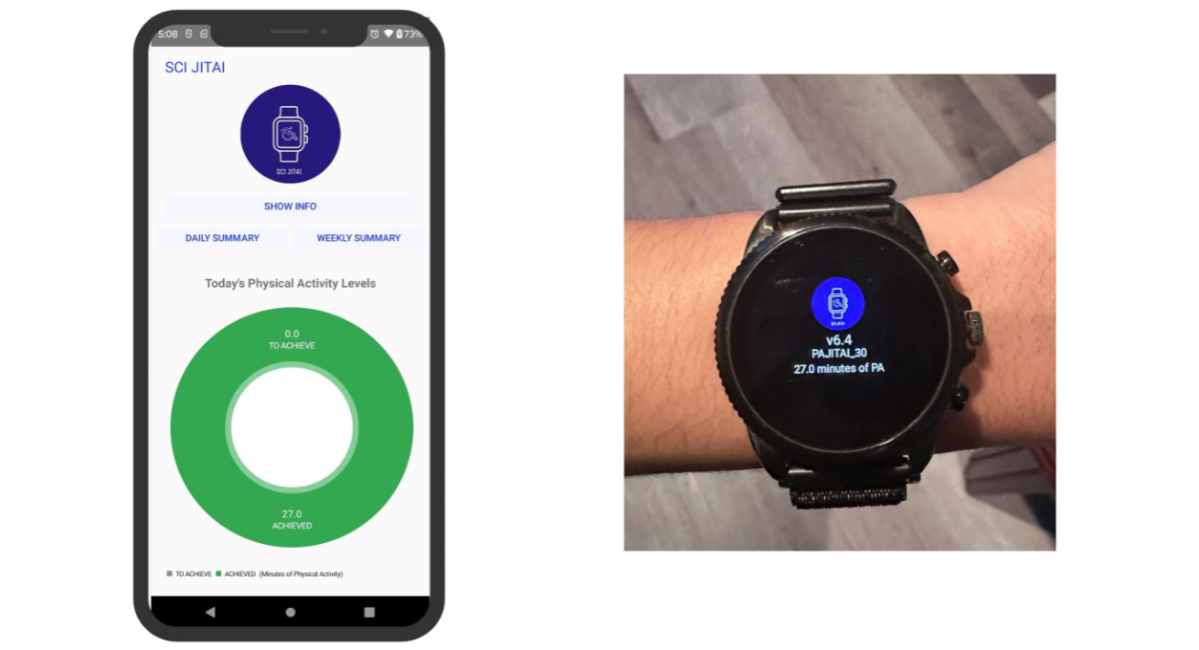
Example of real-time daily feedback on the smartphone (left) and smartwatch (right). The goal for the day in this example is 27 minutes of moderate-intensity physical activity (PA). This is in addition to having access to PA levels performed over the last 7 days on the smartphone.

**Figure 7:**
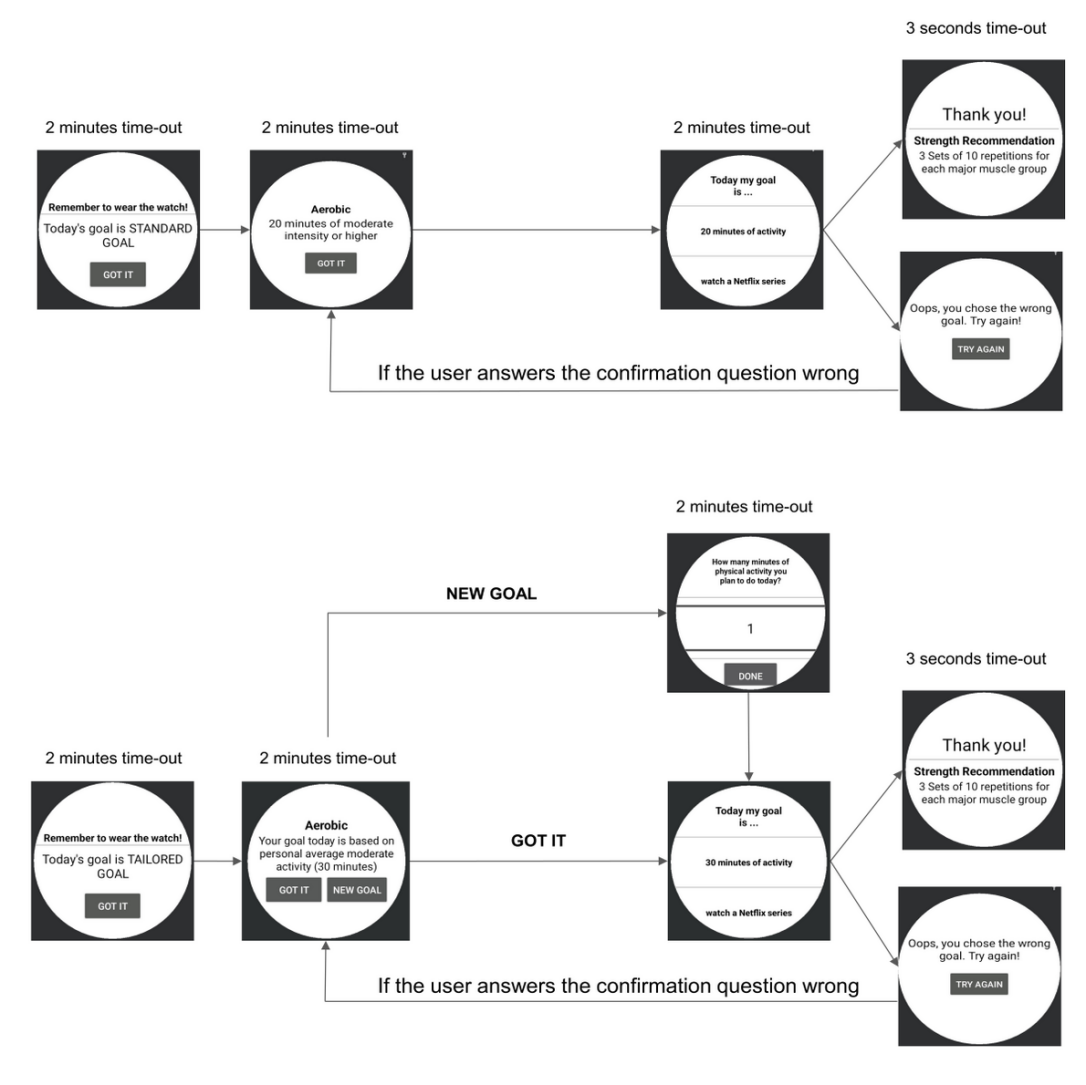
Participants will be randomized to a standard goal, tailored goal, or no goal condition within 1 to 2 hours of waking up. Top: goal setting—standard goal. Bottom: goal setting—tailored goal. The “Try again” screen will automatically time out back to the goal screen if the user does not press the “Try again” button in 3 seconds. Messages will be reprompted up to 3 times.

**Figure 8:**
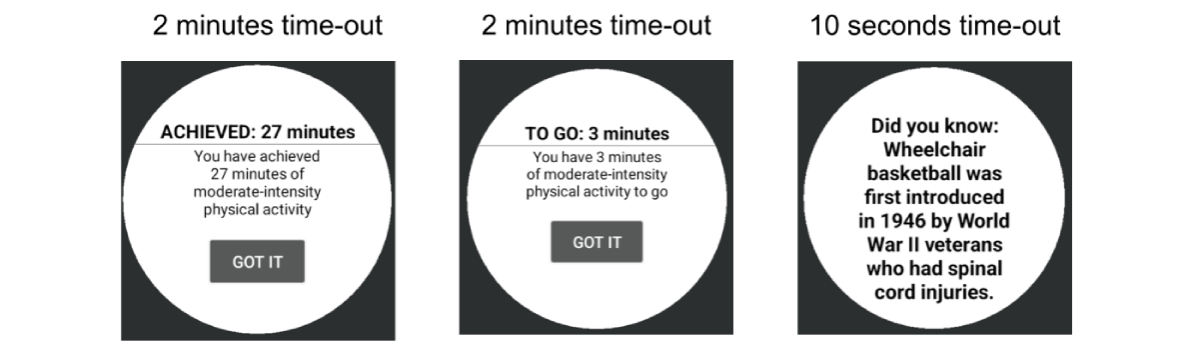
Participants will be randomized to a just-in-time adaptive intervention (JITAI) message or no message twice a day. Left: JITAI message—achieved. Middle: JITAI message—to go. Right: if the user acknowledges the JITAI message, then the user receives a “fortune cookie"–like message on the smartwatch.

**Figure 9:**
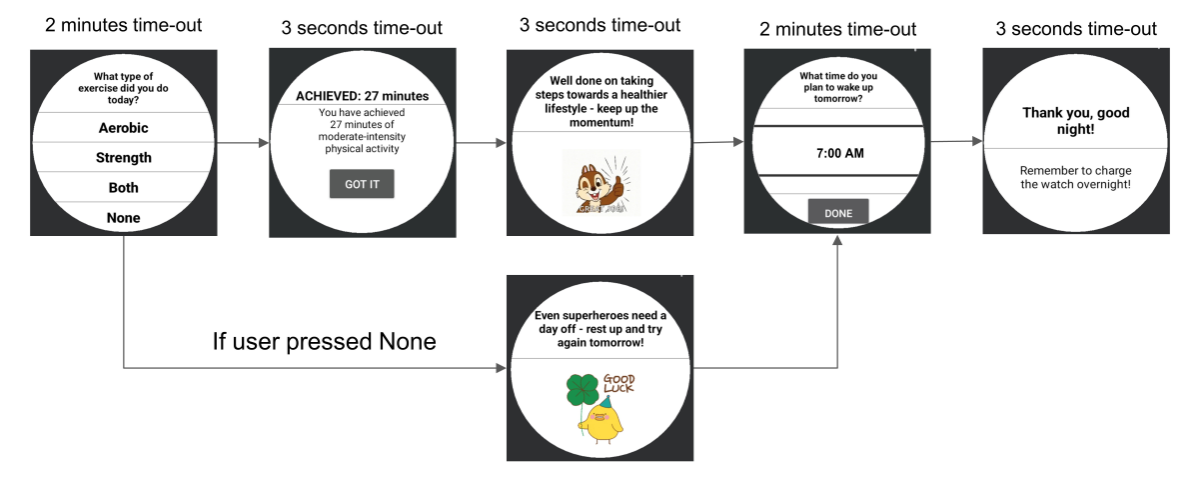
Participants will respond to an end-of-day ecological momentary assessment to confirm whether they performed exercises (aerobic, strength, or both).

During the 22-week intervention period, the PA feedback and recommendation messages will be delivered using microrandomization, which involves random selection of intervention options at each possible time of delivery [29,45]. The microrandomizations will assess the proximal benefits of just-in-time interventions (Figures 5 and 6): (1) *PA recommendations* by randomizing the participant with a 33% probability to a standard, tailored, or no goal within 1 to 2 hours of waking up (Figure 7) and (2) *PA feedback prompts* by randomizing the participant with a 50% probability to a JITAI message or no message twice a day (Figure 8). The first and second PA feedback prompts are randomized between 2 and 4 hours after wake-up and 6 to 8 hours after wake-up, respectively. Table 2 shows the type and number of messages participants will receive during the intervention period. Within the *PA feedback prompts*, the participant will be randomized to an achieved or to-go message with a 25% probability per day. The participant can acknowledge seeing the JITAI message by pressing a “Got it” pop-up button that appears on their smartwatch. Furthermore, since the smartwatch’s accelerometer sensor can detect movement-based PA and not the intensity of the strength exercises, participants will respond to a short ecological momentary assessment, presented on their smartwatch at the end of the day to confirm whether they performed exercises (aerobic, strength, or both; Figure 9). The choice of 2 randomizations per day for *feedback prompts* was based on our pilot study [24], in which the participants felt that 5 interruptions per day were reasonable and not distracting. The other 3 interruptions include goal-type message, WI reminder, and a short ecologic momentary assessment at the end of the day.

**Table 2:** The type and average number of messages participants will receive during the physical activity intervention period.

Goal-type message (number of days in a week)	Number of JITAI^a^ or no message per goal type (randomized)		Number and type of JITAI messages per goal type (randomized)		Number of ecological momentary assessments per goal type
	JITAI message	No message	JITAI (achieved)	JITAI (to go)	
Standard goal (2)	2	2	1	1	2
Tailored goal (2)	2	2	1	1	2
No goal (2)	2	2	2	0	2
Day off (1)	0	0	0	0	1

^a^JITAI: just-in-time adaptive intervention.

#### Personalized PA Threshold Identification

**Figure 10:**
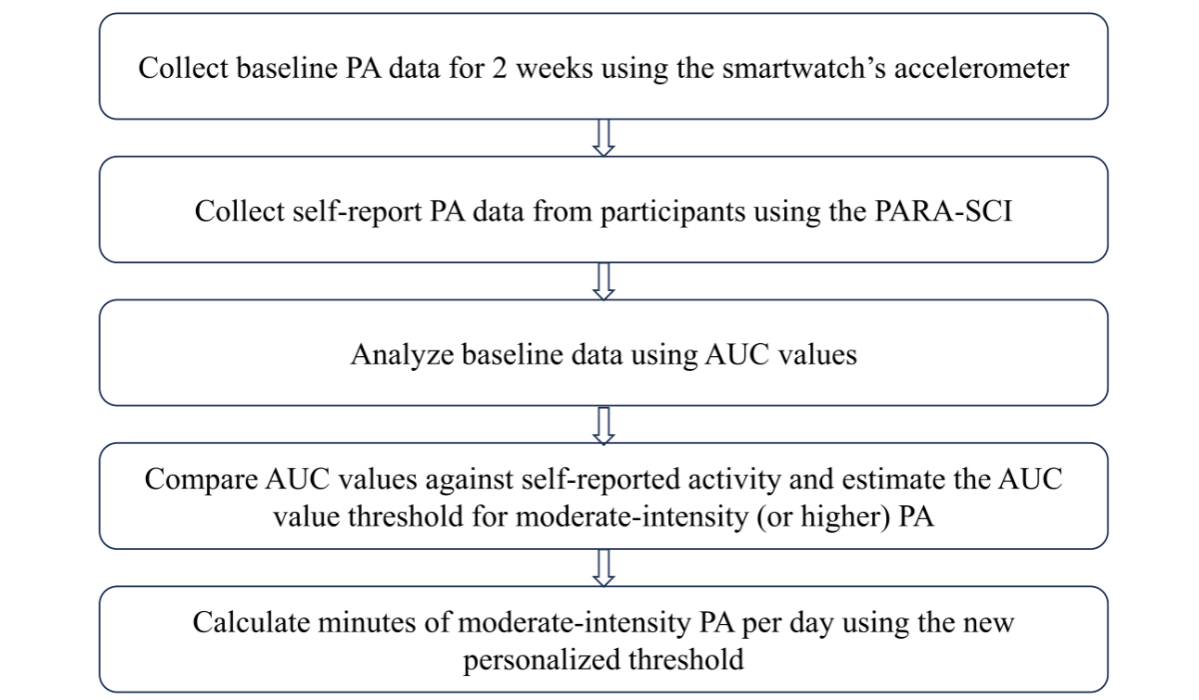
Flowchart to identify a personalized physical activity (PA) threshold. The new threshold value will allow us to calculate minutes of moderate-intensity PA per day for each participant. AUC: area under the curve; PARA-SCI: Physical Activity Recall Assessment for people with spinal cord injury.

A multifaceted approach of using self-reported PA data with sensor data from the smartwatch during the baseline period will help identify personalized PA thresholds. These personalized PA thresholds are then used by the JITAI to determine whether minutes of moderate-intensity PA per day for each participant have been achieved each day for the rest of the study period. Baseline PA data are quantified using a motion summary measure that is computationally feasible on the smartwatch for real-time computation. Area under the curve (AUC) values obtained from high-pass–filtered triaxial accelerometer signals are summed each minute to obtain a single motion measure per minute, which is then further summed throughout the day. The AUC values provide a quantitative and cumulative measure of PA over time because they integrate the intensity of motion activity over time. The choice of a relatively simple measure of PA intensity is due to its low computational demands on the central processing unit, causing a reduced impact on the battery life of the smartwatch. In addition, self-reported PA data are collected through the Physical Activity Recall Assessment for people with SCI, which provides subjective insights from the participants [46]. After the baseline data collection, we will meticulously compare the self-reported PA data with AUC data to determine a new threshold value for each participant (Figure 10) to gain a more comprehensive understanding of their actual PA levels and intensity. The rationale for this process is due to various functional deficits resulting from complete or incomplete SCI at different neurological levels (cervical, thoracic, lumbar, and sacral) [47]. These functional deficits, in turn, lead to large biomechanical variations of upper extremity use during exercise and mobility. Moreover, developing person-specific models that are trained to recognize the types of activities each person with SCI can perform would be burdensome. Our approach of using movement data from a wrist-worn smartwatch allows us to generalize the intervention to persons with SCI without requiring them to come to a laboratory for extensive measurements.

### Sample Size Determination

Sample size calculation for the primary aim (a 2-arm randomized controlled trial) indicated that 82 participants are needed per group to detect at least a standardized mean difference of 0.44 [48] (considered small to moderate) in the primary outcome, with 80% power (assuming a 2-sided test with an α level of .05). On the basis of prior research [49,50] and our pilot study [24], we anticipate a 20% dropout rate. Thus, we plan to recruit a total of 196 participants with SCI (98 participants per group). In addition, power analysis for the secondary aim (an MRT) based on 2 randomizations per day indicated that a sample size of 69 is needed to detect a difference between JITAI message and no message on the proximal PA achieved for the WI+JITAI arm [30,51]. We assumed an average proximal treatment effect for message versus no message of 0.10 (considered small) that starts with 0.12 (ie, a small effect) and decreases linearly for the 16 weeks, an α level of .05, and 80% power [30]. We will have sufficient power for the MRT given we plan to recruit 98 individuals for the WI+JITAI arm to ensure that the primary aim is powered.

### Recruitment

Our collaborative research team will work with coinvestigators from Magee Rehabilitation Hospital, MossRehab Hospital, Thomas Jefferson University Hospital, and Good Shepherd Rehabilitation Network to recruit participants for this study. We have an established collaboration for the past 6 years. Participants will be recruited through a multipronged approach including (1) the clinicians identifying patients with SCI at their clinics who are eligible for the study and providing them with study flyers, (2) flyers being placed in the common areas of the outpatient clinics, and (3) flyers being distributed through their listserves. In addition, we will send flyers to local and national support groups for individuals with SCI in the United States to increase the visibility of our study.

### Eligibility Criteria

Participants will be included if they (1) are aged 18 to 75 years; (2) have a traumatic (neurological level of injury at cervical level 5 and below) or nontraumatic SCI; (3) are ≥6 months after SCI; (4) use a manual or a power wheelchair as their primary means of mobility (>80% of time); (5) show readiness for PA as assessed by the Physical Activity Readiness Questionnaire; (6) can use their upper arms for exercise; (7) can use a smartphone including interacting, recharging, and carrying it with them; and (8) can use a smartwatch including interacting, recharging, and wearing it. Participants will be excluded if they (1) have any secondary complications such as pressure injuries, contractures, and infections that medically restrict their activity in any way; (2) are diagnosed with traumatic brain injury; and (3) have a history of cardiovascular disease.

### Screening

Participants who contact us and indicate an interest to participate in the study will be informed about the inclusion and exclusion criteria. Participants will verbally indicate if they meet the criteria. If they meet the criteria, we will schedule a first meeting with them. If they do not qualify, we will not record or store any data.

### Protocol and Assessments

#### Participant Meetings

All study procedures will be performed at Temple University or in the community. Our team can work with the participants remotely via Health Insurance Portability and Accountability Act–compliant video conference meeting software, such as Zoom (Zoom Video Communications), to obtain study-related information; we will send participants an Android-based smartphone and a smartwatch through postal mail. The research team can also meet with participants for a 30-minute face-to-face meeting if they prefer and are located within the greater Philadelphia region. All precautions will be taken to minimize the risks to participants and the research staff during the face-to-face meeting. Recruiting individuals who wish to remotely participate in the study will help improve the chances of including individuals with SCI who face transportation barriers, boost recruitment, and enhance retention.

The initial meeting will allow individuals with SCI to provide informed consent and answer questions about their demographics, SCI, wheelchair, and health and activity history. From prior research studies, the investigators estimate that the questionnaires will take approximately 60 to 90 minutes to complete (Textbox 2). Participants will also answer questionnaires 4 to 12 indicated in Textbox 2 at the end of weeks 2, 8, 16, and 24.

**Questionnaire answered by the participants at the initial meeting (numbers 1-12) and at the end of weeks 2, 8, 16, and 24 (numbers 4-12).**

**Questionnaire**

1. Demographics (eg, age, biological sex, gender, race, ethnicity, and marital status)

2. Spinal cord injury (SCI) and level of injury (SCI Core Data Set Form, SCI Spinal Column Injury Basic Data Set Form, Non-traumatic SCI Data Sets Version 1.0 Form, and American Spinal Cord Injury Association Impairment Scale)

3. Mobility (Assistive Mobility Devices and Orthoses Form)

4. Pressure injuries (Pressure Ulcer Scale for Healing Tool 3.0), if applicable

5. Pain (International SCI Pain Basic Data Set Version 2.0 Form)

6. Fatigue (Neuro-QOL Item Bank Version 1.0 Fatigue Short Form)

7. Function (Spinal Cord Independence Measure III)

8. Quality of life (Quality of Life Basic Data Set Version 1.0)

9. Physical activity (PA) history (Physical Activity Recall Assessment for people with SCI Survey and Leisure Time Physical Activity Questionnaire for People with SCI Survey)

10. PA behavior (SCI Physical Activity Behavior Survey)

11. Barriers to PA (Barriers to Physical Activity Questionnaire for People with Mobility Impairments)

12. Participants’ motivation (Physical Activity and Leisure Motivation Scale)

#### Allocation and Blinding

After the baseline data collection, participants will be randomly allocated to one of 2 study arms (WI arm or WI+JITAI arm) based on the randomization schedule established by the project biostatistician. Participants will be block randomized on the basis of their level of injury: tetraplegia (higher-level injury) versus other (lower-level injury).

Participants will be masked (single blinding) to the study arm. All participants will receive the same WI program and commercial off-the-shelf technology. Participants in both arms will have access to minutes of moderate-intensity PA performed over the last 7 days, which is designed to motivate them to use the smartwatch in the community. In addition, the participants in the WI+JITAI arm will receive randomized prompts of various types of tailored feedback and PA recommendations over the course of the study.

#### Participant Testing in the Community

##### Overview

The research team will conduct a web-based training session for all participants to introduce them to the WI program, and study-related smartphone and smartwatch. Participants will be loaned an Android-based smartphone and smartwatch. Participants will be asked to wear the smartwatch every day for ≥12 hours. Participants are expected to wear the smartwatch for at least 6 days of the week to assist with good quality data collection for the study. They will be asked to charge the smartphone and smartwatch nightly. Participants can leave the smartphone at home. Once smartphone and smartwatch setup is complete, participants will be instructed to continue with their normal daily routine for 2 weeks during baseline data collection. With regard to the WI and WI+JITAI arms, at the end of the study, the research team will send the participants a return postal packet for them to return the study-related smartphone and smartwatch.

##### WI Program

Participants will access the 14-week WI program. Participants will receive reminders on the smartwatch to access the WI program. Furthermore, the study team will emphasize features such as badges for completing weekly modules to promote engagement with the WI program.

##### WI Arm

Participants in the WI arm will take part in the WI program mentioned above. They will also have access to the minutes of moderate-intensity PA performed over the last 7 days on their study smartphone. After the WI program is completed in week 16, the participants will transition to the PA sustainability phase (weeks 17-24), which will include participants having continued access to the WI program and PA performed over the last 7 days.

##### WI+JITAI Arm

Participants in the WI+JITAI arm will also take part in the WI program. They will have access to minutes of moderate-intensity PA performed over the last 7 days on their study smartphone, just-in-time feedback, and PA recommendations that are informed by PA guidelines for adults with SCI [43] on their smartwatch, and near–real-time minutes of PA performed over the day on their smartwatch. After the WI program is completed in week 16, the participants will transition to the PA sustainability phase (weeks 17-24), which will include participants having continued access to the WI program and feedback and recommendations on their study smartphone and smartwatch.

#### Compensation

Participants will receive gift cards for a total value of US $160 for completing the study. The participant incentives are provided upon completion of meetings and data collection. The incentives are divided into US $20 per meeting for the initial and final meetings of the study and US $20 for every 4-week duration of the study for a 24-week study.

### Statistical Analysis

#### Overview

For all aims, descriptive statistics will be obtained to assess distributional assumptions, and any transformations will be applied if needed. In addition, we will examine patterns of missingness and handle this appropriately through either multiple imputation or using models that can appropriately handle missingness (eg, mixed-effects models). We will also examine whether there are any group differences on baseline covariates (eg, pain, fatigue, health, and activity history) despite randomization, and if so, we will statistically control for these variables in our models. All analysis will be performed with an α level of .05.

#### Primary Aim Analysis

We hypothesize that the integration of WI+JITAI will produce significantly higher PA levels over 14 weeks than the standard WI alone. Exploratory outcomes include long-term PA (measured via sensors and self-report) over 24 weeks (approximately 6 months). To test our hypothesis, we will fit a mixed-effects model in which the group indicator variable is a predictor, and the outcomes are the PA measurements at 2, 8, and 16 weeks. We will include an interaction with time in the model because we hypothesize that although PA levels may increase in both groups, they will increase more in the WI+JITAI group. We will also include a random intercept and slope for time. To evaluate long-term PA, we will include the PA levels at 24 weeks in the model.

#### Secondary Aim Analysis

Data from the individuals with SCI assigned to the WI+JITAI condition will be used to assess the impact of randomly provided just-in-time PA feedback on proximal PA. The analysis will address the question of whether, on average, the just-in-time PA feedback intervention has a proximal effect on moderate-intensity PA within 120 minutes of the feedback prompt. We will also assess the impact of randomly provided goal-type message for the day on daily PA level achievement. We will use a generalization of regression analysis specifically developed to ensure unbiased estimation of causal effects of time-varying treatments (eg, feedback prompts) in mHealth settings [52,53]. Since the treatment is time-varying, potential time-varying moderators, such as pain or fatigue, and potential control variables, such as PA in the 30 minutes before the randomization decision point, may be outcomes of past treatment. Because our randomization probability is constant over the course of the study, we can use generalized estimating equations in which we include availability as weights with an independence working correlation matrix and sandwich SEs as described in Boruvka et al [52]. We use an independence correlation matrix because causal effect estimates are biased if off-diagonal elements are present [48,54]. These analyses pool time-varying, longitudinal data across all study participants. There are 2 possible intervention options at each decision point (PA feedback or no feedback). Thus, the models will include an indicator variable for PA feedback versus no feedback. In addition, we will include day in the study, as well as the interaction between day and each treatment indicator variable, and potential control variables such as PA in the 30 minutes before the randomization decision point to assess whether the proximal effect varies over the course of the study.

#### Exploratory Analysis

Moderators will include age, biological sex, race, ethnicity, level of injury, function, mobility, pain, and fatigue. The exploratory analysis aims to identify subgroups of individuals with SCI who are likely to benefit the most from the integration of just-in-time PA feedback and recommendations with WI. We will conduct 2 moderation analyses. First, we will incorporate moderators into our primary aim models to identify subgroups of individuals with SCI who are likely to benefit the most from the integration of just-in-time PA feedback and recommendations with WI. Second, we will address potential time-varying moderators, such as prior PA and engagement with the JITAI, self-reported pain (number and type), and fatigue, which can be incorporated into the model described in the secondary analysis [52]. Engagement with JITAI will be captured by participants’ response to the “Got it” pop-up button that appears on the smartwatch when they receive a JITAI message. We will also examine potential time-invariant moderators such as biological sex and SCI level. This analysis will allow us to identify subgroups of individuals who are likely to benefit from particular types of messages (eg, tailored vs standard messages).

## Results

### Overview

This study was funded by the Eunice Kennedy Shriver National Institute of Child Health and Human Development, National Institutes of Health (Multimedia Appendix 1). Recruitment and enrollment began on May 25, 2023. As of the submission of this manuscript, participant enrollment is ongoing. Since the research study involves no more than minimal risk, there will be no interim analysis, and data and safety monitoring will occur in accordance with guidelines by the Eunice Kennedy Shriver National Institute of Child Health and Human Development and the institutional review board of record. Data analysis is expected to be completed within 6 months of ending participant data collection.

### Dissemination Policy

We plan to disseminate our findings in the form of interdisciplinary peer-reviewed manuscripts, presentations at local rehabilitation hospitals, and presentations at national and international conferences.

## Discussion

### Implications

PA interventions paired with mHealth technologies that continuously measure behavior can provide novel insights about PA patterns in the community. Individuals with SCI, although generally stable in the chronic postrehabilitation phase, are at a higher risk than the general population for secondary health conditions due to physiological changes associated with injury and due to sedentary behaviors potentially related to the use of assistive technology such as wheelchairs [50,55]. Accurate, real-time measurement of PA using affordable and convenient wearable monitors will not only enable more research on existing behavior and relationships to health outcomes but also allow insight into innovative JITAIs that may help individuals with SCI become more active in everyday life. To address these needs, we will investigate whether combining a JITAI with a WI program increases and sustains PA levels among individuals with SCI in the community. The JITAI will automatically detect PA levels and provide behavior-sensitive PA recommendations and feedback to increase PA in individuals with SCI. Although existing PA-based programs often require individuals to participate in them several times a week, a JITAI can act as a constant companion that encourages individuals and assists them in attaining their daily and weekly PA goals. The JITAI will operate as people go about their everyday lives in the community; as they do so, the JITAI will provide timely and tailored encouragement, feedback, and PA interventions designed to support sustainable behavior change [24,27].

Regular PA feedback has the potential to make individuals self-aware of their PA levels, which may be effective in a small percentage of individuals who are motivated to increase their PA levels [56]. To achieve long-term PA performance required to support behavior change and maintenance, there is a need to tailor PA feedback and recommendations based on an individual’s behaviors and goals. This study will focus on understanding the impact of different messages on promoting proximal PA performance. Furthermore, use of an MRT study design will allow us to assess the effect of the JITAI components on participants’ PA levels over time. MRT designs enable us to investigate the causal proximal effects of just-in-time interventions and test time-varying moderators of those effects [29,30]. On the basis of the impact of the type of messages on proximal PA, MRT results will allow us to determine the relative utility of various feedback and PA recommendation strategies.

### Anticipated Findings

This project will investigate a state-of-the-art JITAI-enhanced WI program, which is guided by the COM-B model [31], that highlights the importance of capability, motivation, and opportunity for behavior change. JITAIs using mHealth technology can deliver an intervention that feels dynamic and personally responsive and thus more engaging to the individual. In this study, we will test whether PA levels change with WI and JITAI-based PA recommendations and feedback prompts. We will test 3 ways of providing PA recommendations (standard goal, tailored goal, or no goal) and 3 types of feedback prompts (PA achieved, PA to-go, or PA feedback not presented) to determine whether adding the JITAI to the WI may impact PA levels and sustain changes over time. Furthermore, the study design will allow us to identify subgroups of individuals with SCI who are likely to benefit the most from the integration of JITAI with WI. The identification of such subgroups will inform the design, development, and evaluation of JITAIs for future physical rehabilitation intervention studies for individuals with and without disabilities.

### Limitations

One of the potential limitations of this study is that our JITAI has been developed for Android-based smartphones, which provide greater flexibility in research and are used by 53.9% of smartphone users in North America [57]. In addition, we are loaning participants study-related devices that are not personal devices. If we can show the efficacy of the JITAI with this randomized clinical trial using Android devices, the approach of using a JITAI to provide a PA intervention could be converted to other operating systems (eg, iPhone with Apple Watch). This step-wise approach is cost-effective and will test the viability of the underlying behavior change approach; future versions of the JITAI that use personal devices may further improve compliance and reduce burden. We will recruit adults with SCI between 18 and 75 years old; this age range includes 89.2% of all those with SCI [2]. Children will not be studied due to their use of different wheelchairs during their development, and because children’s responses to and recovery from an SCI differ from those of adults. However, evaluation of JITAI in children with SCI is a potential avenue for future investigations. Another limitation concerns the use of a relatively simple measure of PA intensity. Future work might use more sophisticated, person-specific models trained to recognize the types of activities each person with SCI can perform when obtaining moderate-intensity PA.

### Conclusions

The JITAI has the potential to improve long-term PA performance by delivering tailored just-in-time feedback based on the person’s actual PA behavior rather than a generic PA recommendation. New insights from this study may assist intervention developers in creating more engaging PA interventions for individuals with disabilities. Our work could lead to more impactful and sustainable clinical and rehabilitation interventions for this population by demonstrating how the use of affordable, off-the-shelf consumer devices to create a JITAI may enhance existing interventions.

## Appendix

Multimedia Appendix 1 Peer-review report by the CMPC - Clinical Management of Patients in Community-based Settings Study Section - National Institutes of Health (NIH), United States.

## Abbreviations

AUC: area under the curve
COM-B: capability, opportunity, and motivation model
JITAI: just-in-time adaptive intervention
mHealth: mobile health
MRT: microrandomized trial
NCHPAD: National Center on Health, Physical Activity, and Disability
PA: physical activity
PARA-SCI: Physical Activity Recall Assessment for people with spinal cord injury
SCI: spinal cord injury
WI: web-based physical activity intervention

## Data Availability

Data sharing is not applicable to this paper because no data sets were generated or analyzed during development of this study protocol. The deidentified data sets generated during the actual research study will be presented in the main manuscript and as additional supporting files.
