# Supplementary material for: mHealth-Based Just-in-Time Adaptive Intervention to Improve the Physical Activity Levels of Individuals With Spinal Cord Injury: Protocol for a Randomized Controlled Trial
Source: JMIR Res Protoc. 2024 Jun 28;13:e57699. doi: 10.2196/57699 (PMC11245659; doi:10.2196/57699)
Supplement: Multimedia Appendix 1 [file resprot_v13i1e57699_app1.pdf]

SUMMARY STATEMENT

PROGRAM CONTACT: (Privileged Communication) *Release Date:* 06/23/2020
Revised Date:

Application Number: 1 R01 HD103904-01

Principal Investigator

HIREMATH, SHIVAYOGI VISHWANATH

Applicant Organization: TEMPLE UNIV OF THE COMMONWEALTH

Review Group: CMPC
Clinical Management of Patients in Community-based Settings Study Section

Meeting Date: 06/08/2020
Council: OCT 2020
Requested Start: 09/01/2020
RFA/PA: PA18-480
PCC: NCMRR-TC

Project Title: mHealth-based Just-In-Time Adaptive Intervention to Improve Physical Activity Levels of Individuals with Spinal Cord Injury
SRG Action: Impact Score:28 Percentile:15
Next Steps: Visit https://grants.nih.gov/grants/next_steps.htm
Human Subjects: 30-Human subjects involved - Certified, no SRG concerns
Animal Subjects: 10-No live vertebrate animals involved for competing appl.
Gender: 1A-Both genders, scientifically acceptable
Minority: 1A-Minorities and non-minorities, scientifically acceptable
Age: 3A-No children included, scientifically acceptable

ADMINISTRATIVE BUDGET NOTE: The budget shown is the requested budget and has not been adjusted to reflect any recommendations made by reviewers. If an award is planned, the costs will be calculated by Institute grants management staff based on the recommendations outlined below in the COMMITTEE BUDGET RECOMMENDATIONS section.
EARLY STAGE INVESTIGATOR
NEW INVESTIGATOR

1R01HD103904-01 Hiremath, Shivayogi

EARLY STAGE INVESTIGATOR
NEW INVESTIGATOR

RESUME AND SUMMARY OF DISCUSSION: This application proposes to evaluate the integration of a mobile health just-in-time adaptive intervention to increase and sustain physical activity levels among individuals with spinal cord injuries (SCI). Reviewers described this application as significant for its evaluation of the varying algorithms that are used to classify activity levels across commercially available devices. Measuring activity levels accurately with these devices has posed significant issues for wheelchair users given that this output is based on ambulatory data. This project is led by a highly qualified research team with a history of collaboration and prior publications. The monitoring and use of real time activity sensor data over long periods of time is innovative. Although the targeted enrollment is considered large, the strong collaborations between the project team and two large spinal cord injury rehabilitation centers makes this feasible. The use of a micro-randomized trial (MRT) within the larger randomized trial provides a means to target specific secondary questions gauging how the feedback is delivered to the study participants. The outcome measures are rigorous and evidenced-based and include the use of SCI Common Data Elements. Reviewers also discussed minor weaknesses in the approach, specifically study rigor is a potential concern given there is no justification for the estimated effect size for calculating the same size in both the overall RCT and the embedded MRT based on the pilot data presented. Overall, this significant application will have a high impact on the promotion and sustainability of physical activity programs for individuals with spinal cord injuries.

DESCRIPTION (provided by applicant): The lack of regular physical activity (PA) in over 290,000 individuals with spinal cord injury (SCI) in the United States (US) is an ongoing health crisis. This lack of activity has potentially devastating consequences because low levels of PA in people with SCI elevates the risk of mortality due to cardiovascular diseases, diabetes, and lung disease. Furthermore, low levels of PA in individuals with SCI have been associated with secondary conditions such as pain, fatigue, weight gain, and deconditioning. Regular PA and exercise-based interventions have been linked with improved outcomes and healthier lifestyles among those with SCI. Sensor- based activity monitors can assess PA and exercise interventions by quantifying wheelchair movement, movement of the individual, and physiological changes. However, these monitors do not provide real-time, tailored feedback and recommendations that might help individuals with SCI increase their PA levels in the community. The overarching goal of this proposal is to evaluate a sensor-enabled, just-in-time adaptive intervention (JITAI) strategy to increase and sustain PA levels among individuals with SCI in their communities. The long-term goal of this research is to effectively integrate a mobile health JITAI with existing PA intervention programs to motivate health-related behavior change in individuals with SCI. A primary objective of this proposal is to extend our pilot work to evaluate the integration of a JITAI with a web-based 14-week PA intervention program from the National Center on Health, Physical Activity and Disability (Aim 1). We hypothesize that the integration of web-based PA intervention program with JITAI will result in significantly higher PA levels over 14 weeks compared to the standard we-based PA intervention program alone. A secondary objective of this study is to extend existing algorithms that use commercial wearable technology to robustly detect PA behaviors to facilitate the delivery of tailored just-in-time actionable feedback and PA recommendations for individuals with SCI (Aims 3 and 4). The integration of the JITAI, which provides feedback and PA recommendations due to sensor-based assessments of PA, with the standard web-based PA intervention program will be tested via a clinical trial that combines a randomized controlled trial and a micro-randomized trial. Our team includes investigators with expertise in SCI research, mobile health, PA tracking, and behavioral change interventions. The proposed study will yield novel insights about JITAIs and JITAIs combined with more traditional, web-based PA intervention programs, which will help researchers design engaging PA

interventions for individuals with disability in the community that may improve their health and quality of life.

PUBLIC HEALTH RELEVANCE: The lack of regular physical activity in over 290,000 individuals with spinal cord injury (SCI) in the US is an ongoing health crisis. The proposed study evaluates the integration of a just-in-time-adaptive intervention strategy with a web-based physical activity intervention program for individuals with SCI in the community.

CRITIQUE 1

Significance: 1
Investigator(s): 1
Innovation: 1
Approach: 1
Environment: 1

Overall Impact: This proposal addresses the very significant issue of physical inactivity in the wheelchair-using SCI population and overall impact is high because the research is likely to provide a critical link in improving physical engagement through the use of personalized and targeted feedback based on the person's own movement patterns. This project is highly innovative in the use of JITAI that integrates the movement data from the wearable sensors and this has not yet been accomplished for this population. The approach is very strong, utilizing a randomized controlled trial with a significant number of participants. The only weakness, which is mild and acknowledged by the investigators, is the exclusion of power wheelchair users who may also benefit from this work. The investigator is well-trained and has significant experience in the use of JITAI and the processing of real-time movement data from wearable sensors. The PI has collaborated with all of the investigators previously with demonstrated productivity. In conclusion, the overall impact of this proposed project is exceptional because it will advance interventions to increase engagement in physical activity in wheelchair users with spinal cord injury, with potential impact on reducing secondary conditions.

1. Significance:

Strengths

- Physical activity participation by the SCI population is critical for prevention of several secondary conditions across the lifespan, so the results of this study would have high impact if shown to be effective in increasing physical activity.
- This study will also address the problem of varying algorithms for classifying activity levels across commercially available devices (because they are based on ambulatory data), which has posed a significant issue for accurately measuring activity levels with off-the shelf activity monitors for wheelchair users.
- This engages the expertise of behavior change experts in the delivery of the mHealth intervention and use of an evidence based approach (JITAI) to individualize the feedback; if effective, this could significantly improve activity engagement by the participants.
- This study will help bridge the gap between use of sensors by research labs (which requires significant pre-processing and classification) and clinicians or individuals with SCI who want to accurately track activity levels without needing to learn how to use the pre-processing and algorithm/classification techniques.

Weaknesses

- No weaknesses noted.

2. Investigator(s):

Strengths

- Dr. Hiremath’s experience with activity monitoring and activity classification using wearable sensors in wheelchair users with spinal cord injury is evident. He also has several years experience developing Just In Time Adaptive Interventions for this population.
- Dr. Hiremath has collaborated with all of the Co-investigators extensively on past projects and there are multiple publications between them and including many of them together.
- Dr. Hiremath is PI of a current NIH study (SCI related) and has successfully led as PI on a foundation grant (SCI-related).
- The study team brings a wealth of experience and complementary areas of expertise to this project.

Weaknesses

- No weaknesses noted. This appears to be a well-functioning team with demonstrated ability to collaborate productively.

3. Innovation:

Strengths

- This study will be the first to introduce a JITAI feature to an mHealth intervention for the SCI population to target physical activity engagement.
- Monitoring and using (in real time) activity sensor data over very long periods of time (24 weeks in this study) has not been reported previously; other studies have monitored the data and uploaded it at the end of the study to process.
- This study provides a comparison between web-based interventions (which aren’t all that novel, but are becoming more popular as more people stay home) and use of JITAI with a web-based intervention which individualizes the experience. This has not yet been compared in the SCI population.

Weaknesses

- No weaknesses noted.

4. Approach:

Strengths

- The JITAI proposed as an extension to the web-based activity has been developed and pilot testing completed by the PI over the past 5 years with results that support the next step of adding it to the web-based activity for comparison.
- This study employs a micro randomized trial within a larger randomized trial, providing a way to target specific secondary questions around how the feedback is delivered to the participants.
- The targeted enrollment is large, but feasible given the strong collaborations with two large SCI rehabilitation centers.

- The outcome measures are rigorous, evidence-based, and include use of the SCI Common Data Elements.
- The physical activity guidelines are based on evidence for the SCI population.

Weaknesses

- As acknowledged by the investigators, a weakness is the exclusion of power wheelchair users who would also benefit from physical activity; some power wheelchair users have similar injury levels as those in manual wheelchairs and could possibly perform similar exercise programming. It will be important to consider this demographic because it is known that as individuals with SCI age, they are more likely to shift from a manual wheelchair to a power wheelchair. (Not score driving)
- Weak/minor weakness, not score driving: Exclusion of participants with any active wounds may be restrictive and result in lower enrollment as literature indicates up to 35% of individuals with SCI may have a pressure injury at any given time. Investigators may want to consider loosening this restriction to allow earlier stage injuries (Stage I or II) to be present.

5. Environment:

Strengths

- The research environment for the project is well-equipped at all sites. Dr. Hiremath has developed considerable resources around activity monitoring data analysis and data storage, which will be critical for this large clinical trial. All of the university environments support this project through ample laboratory space and office space and equipment.
- Moss Rehab's Spinal Cord Injury Center cares for 200 new and 500 chronic SCI patients each year, which will support recruitment activities.
- Thomas Jefferson University is affiliated with Magee Rehabilitation, one of the 12 Model SCI Centers and the only one in the region where this study will take place, providing access to another 275 acute SCI patients and also to patients in the Lifetime Follow Up System of Care.

Weaknesses

- No weaknesses noted.

Study Timeline:

Strengths

- The timeline appears feasible for conducting the activities in this study as long as recruitment/enrollment is successful.

Weaknesses

- None noted.

Protections for Human Subjects:

Acceptable Risks and/or Adequate Protections

- The study team will have frequent contact with the participants and strong clinical support available to address challenges that might arise, specifically around pressure injuries.

Data and Safety Monitoring Plan (Applicable for Clinical Trials Only):

Acceptable

- The data safety monitoring plan is detailed and considers the protection of health information delivered via mobile phone app.

Inclusion Plans:

- Sex/Gender: Distribution justified scientifically
- Race/Ethnicity: Distribution justified scientifically
- For NIH-Defined Phase III trials, Plans for valid design and analysis:
- Inclusion/Exclusion Based on Age: Distribution justified scientifically
- There are fewer women with SCI and this has been appropriately reflected in the recruitment plan.

Vertebrate Animals:

Not Applicable (No Vertebrate Animals)

Biohazards:

Not Applicable (No Biohazards)

Resource Sharing Plans:

Budget and Period of Support:

Recommend as Requested

CRITIQUE 2

Significance: 4
Investigator(s): 2
Innovation: 3
Approach: 6
Environment: 3

Overall Impact: This R01 application aims to evaluate an mHealth-based just-in-time adaptive intervention to improve physical activity in individuals with spinal cord injury. It targets a significant problem and addresses a specific knowledge gap to integrate mHealth intervention for the target population. The multi-disciplinary investigative team is excellent. The proposed concept is innovative. The environment is supportive. A few weaknesses noted in: (1) insufficient rigor from the pilot study to support the proposed research; (2) various issues in study approaches that undermine study rigor. Balancing the strengths and weaknesses, the overall impact of the proposed research will likely be only moderate.

1. Significance:

Strengths

- Targeting a significant problem of physical activity in individuals with spinal cord injury.
- Addressing knowledge gaps in integrating mHealth just-in-time adaptive intervention for the target population.

Weaknesses

- Many aspects of the preliminary data, for example, PA level, reasons of drop out, feedback, and relations between pain/fatigue and PA levels, seem to be inconclusive. The rigor of the pilot study to support the proposed research is a potential concern.

2. Investigator(s):

Strengths

- The multidisciplinary team has expertise in SCI research, mHealth, PA tracking, biostatistics, and behavioral change interventions.
- Many members of the team have collaborated in previous works.

Weaknesses

- None

3. Innovation:

Strengths

- The proposed just-in-time adaptive intervention delivered through mobile apps is innovative.

Weaknesses

- None

4. Approach:

Strengths

- Pilot studies developed the initial version of the technology.
- Study process is clearly described. Data collection will leverage many standard validated instruments.
- Data analytics plan is developed. Sex as a biological variable is considered.

Weaknesses

- Multiple theoretical frameworks (conceptual model of JITAI, COM-B, social cognitive theory, transtheoretical model) are proposed and used for different components of the study. Integration of these models or frameworks, in particular, the human factor component in socio-technical systems, is insufficiently discussed.
- The rationale for using the MRT is insufficiently discussed. It seems the assumption is that a specific message intervention will translate into behavioral change in a short timeframe. But there is no detail provided on whether the pilot study supports this assumption.
- There is no support from the pilot study on the estimated effect size for calculation of the sample size for both the overall RCT and the embedded MRT. Study rigor is a potential concern.

- The performance of the machine learning approach to capture PA was not discussed. With randomized messages, the fidelity of the proposed intervention is a potential concern.
- The intervention expects longtime wearing of smartwatch and close proximity to wheelchair every day during the 24 months' study period. There is insufficient discussion on monitoring and ensuring actual use of the system to review the messages. Usage compliance is potential concern.

5. Environment:

Strengths

- Temple University and the collaborating sites have many clinical, technology, and research resources to support the proposed study.

Weaknesses

- None.

Study Timeline:

Strengths

- Study tasks are mapped to timeline

Weaknesses

- None

Protections for Human Subjects:

Acceptable Risks and/or Adequate Protections

- Protection plan is in place

Data and Safety Monitoring Plan (Applicable for Clinical Trials Only):

Acceptable

- DSMB plan is ok

Inclusion Plans:

- Sex/Gender: Distribution justified scientifically
- Race/Ethnicity: Distribution justified scientifically
- For NIH-Defined Phase III trials, Plans for valid design and analysis: Scientifically acceptable
- Inclusion/Exclusion Based on Age: Distribution justified scientifically
- Inclusion plan is developed

Vertebrate Animals:

Not Applicable (No Vertebrate Animals)

Biohazards:

Not Applicable (No Biohazards)

Resource Sharing Plans:

Acceptable

Budget and Period of Support:

Recommend as Requested

CRITIQUE 3

Significance: 2
Investigator(s): 1
Innovation: 1
Approach: 2
Environment: 1

Overall Impact: This application proposes to extend the investigators previous pilot work on a sensor-enabled, just-in-time adaptive intervention (JITAI) strategy by conducting an RCT comparing the existing physical activity (PA) intervention for persons with disabilities from National Center on Health, Physical Activity and Disability (NCHPAD) to an integrated NCHPAD+JITAI. In addition, the investigators plan to extend existing algorithms that use commercial wearable technology to facilitate the delivery of tailored just-in-time actionable feedback and PA recommendations for individuals with SCI. If successful this application is expected to have a high impact on the science of advancing PA in those with SCI. The rigor of previous studies is strong. The investigative team has strong expertise in the multiple disciplines needed for this study. Many aspects of the study are innovative including technology that tracks physical activity levels and provides real-time feedback to wheelchair users through smartphones (JITAI) and use of a micro-randomized trial (MRT) internal to the intervention arm of the study which will add precision about how and under what conditions feedback should be delivered. The strength of the approach includes the preliminary studies, the RTC design (intervention=NCHPAD's web-based PA program + JITAI; Control NCHPAD only), the 8 week follow-up in both groups to assess sustainability, the power analysis, and the analysis plan including a moderator analysis which can identify subgroups of individuals with SCI who are likely the benefit most for the intervention. The clinical and research environments are outstanding. There are several minor issues in the approach that do not majorly decrease the potential impact of the study.

1. Significance:

Strengths

- Significance of the project is supported by literature documenting that the lack of regular physical activity (PA) in over 290,000 individuals with spinal cord injury (SCI) in the United States (US) is an ongoing health crisis due to the devastating health consequences.
- The importance of this proposal is supported by the rigor of past studies. Past interventions have improved outcomes and led to healthier lifestyles but these interventions are limited by the inability to provide real-time, tailored feedback, and recommendations that might help community-dwelling individuals with SCI increase their PA.

- Review published by Tsang, Hiremath, and others found limited data for the off the-self activity monitors including those custom-made for assess PA in wheel chair users in SCI and home or community settings.
- Some studies have pilot tested and evaluated JITAIs in the general population but no previous studies have developed and evaluated JITAIs to improve PA among individuals with SCI.
- Two frameworks underpin the study. The investigators have adapted from Nahum-Shani (et al., 2018) A *Conceptual Model of the JITAI* that uses mHealth technology to deliver intervention options at appropriate times and contexts to support individuals' health behaviors. The framework components decision points, intervention options, tailoring variables, and decision rules can be individualized to achieve desired behavioral outcomes. Further they designed the evidence-based intervention arm (WI+JITAI) to promote the three components, capability, motivation, and opportunity for engaging in PA in the Behavioral Intervention Model.
- The current proposal is supported by the team's pilot that evaluated a mHealth-based JITAI to track physical activity levels of individuals with SCI and provide them with a behavior-sensitive physical activity intervention. The sensor-enabled mobile JITAI that gave automated feedback was supported as a technology that can be used in the home and community setting to improve physical activity levels of individuals with SCI.
- This proposal is significant as it meets the need in the field of developing technologies to monitor outcomes in real-world settings.

Weaknesses

- Although this study has a high likelihood of advancing the science of behavior change and PA in a very vulnerable population, the cost of the feedback equipment (smart phone, Panobike device, smart watch, and 6 months of telephone data package ($1730 per person) is likely to suppress actual long-term use and impact of the science on clinical care and everyday use.

2. Investigator(s):

Strengths

- The study team includes investigators with expertise in SCI clinical care, research, mobile health, PA tracking, and behavioral change interventions.
- PI, Dr. Hiremath, a rehabilitation science researcher, has extensive experience with the technology proposed. He has established a complex research team which has expertise working with wearable activity monitors, a part of the just-in-time adaptive intervention (JITAI) system and databases to securely collect and store sensor data from JITAI and other data proposed. He has experience in behavior-sensitive physical activity interventions for individuals with spinal cord injury in the community.
- Co-I, Dr. Coffman, research interests are at the interface of statistical methods and public health issues. She has developed and applied methods for mediation with intensive longitudinal data collected from wearable and mobile devices. Dr. Coffman will provide her expertise in biostatistics and randomized control trials design to conduct the proposed research study.
- Co-I Dr. Mary Schmidt-Read is a physical therapist and SCI Program Director and Research Coordinator at Magee Rehab, Philadelphia; Co-I,Dr. Marlyn Ramos-Lamboy, a physiatrist and the Medical Director of the Inpatient SCI Program at MossRehab, Einstein Healthcare Network, Philadelphia and
- Co-I, Dr. Marino, a physiatrist, has expertise in outcomes assessment in clinical trials and neurological and functional recovery after SCI. He has collaborated with Dr. Hiremath examining the impact of admission latency and functional status on post-acute outcomes in individuals with spinal cord injury.

- Drs. Schmidt-Read, Ramos-Lamboy and Marino bring decades of clinical and research expertise in SCI, a history of collaboration with the PI and each will facilitate recruitment at their site.
- Co-I Dr. Intille has expertise in computer sciences and an expert in mHealth technologies aimed at measuring physical activity and providing just-in-time feedback to increase people's PA.
- Dr. Mohanraj Thirumalai, is the Director of Information and Communication Technology Core at the Lakeshore Foundation UAB. He has expertise in providing web-based physical activity interventions to people with various disabilities.
- Dr. Inbal Nahum-Shani, an innovator at the Decision-making lab (d3lab), University of Michigan, has expertise in behavioral theory and novel methodologies to develop adaptive interventions that use ongoing information about the individual to modify the type/intensity/delivery-mode of support.
- The majority of the team has a history of multi-year collaboration (Hiremath, Intille, Coffman, Schmidt-Read, Ramos-Lamboy, Marino) and joint publications (Hiremath, Intille, Coffman, Schmidt-Read, Ramos-Lamboy).

Weaknesses

- None

3. Innovation:

Strengths

- Use of a new physical activity monitor system for wheelchair users which has two components, a gyroscope-based wheel rotation monitor for capturing wheelchair wheel movement, and an accelerometer device worn either on the upper-arm or the wrist to track upper arm or wrist acceleration.
- The technology tracks physical activity levels and provides real-time feedback to wheelchair users through smartphones (JITAI).
- Application extends the investigators pilot work on JITAI to evaluate the integration of JITAI with a web-based 14-week PA intervention program from the National Center on Health, Physical Activity and Disability (NCHPAD).
- The NCHPAD intervention is the most widely recommended online PA program across various disabilities and offers a tailored exercise program for individuals that matches the participant's function. Co-I Thirumalai, the original architect of this program, will customize the WI program with the SCI-specific content for the proposed study.
- For JITA, machine-learning algorithms will use the sensor data to detect not just overall activity level but specific types of PAs in individuals with SCI. This precision will make feedback more useful.
- Use of a micro-randomized trial (MRT) internal to the intervention arm of the study will add precision about *how and under what conditions* feedback should be delivered.

Weaknesses

- None

4. Approach:

Strengths

- Study design is a RCT with two arms, NCHPAD's web-based PA intervention program (WI) or the web-based PA intervention program combined with the JITAI (WI + JITAI).

- Within the WI + JITAI arm, an MRT – a clinical trial design for optimizing mHealth interventions – will be used to micro-randomize participants several times a day to various types of tailored feedback and PA recommendations. The micro-randomizations will be designed to address scientific questions concerning *how and under what conditions* it is best to deliver JIT feedback and recommendations.
- The proposed a 24-week longitudinal study will have 2 weeks of baseline PA monitoring, 14 weeks of PA monitoring and intervention, and 8 weeks of continued PA monitoring to assess PA level sustainability.
- The primary outcomes will be assessed at 16 weeks, the exploratory outcome of long-term PA will be assessed over the duration of the study (24 weeks).
- Moderation analysis (age, gender, race/ethnicity, level of injury, function, mobility, pain, and fatigue) which will identify if specific subgroups are likely to benefit the most from the integration of JITAI-based feedback and PA.

Weaknesses

- JITAI has been developed for Android-based smartphones. Although they provide greater flexibility in research and are used by 51% of smartphone users in the US, if the study is successful the lack of availability for over 40% of adults with disability or the issues in transferring the technology to other systems may limit real-world use, at least in the short-term.
- There is no information (from the pilot study) on the potential effect size for calculation of the sample size for both the overall RCT and the embedded MRT.
- The investigators propose longitudinal study that requires wheel chair users to wear both chair and wrist data monitors. No data to support that participants will wear these devices for a prolonged period of time.
- More information (with examples) for the tailored messages would assist in understanding how this component of the intervention would occur. Detailing the potential frequency of different tailored messages, the content of these messages, and how participants might responded to them would be helpful.

5. Environment:

Strengths

- Three clinical sites will be used for recruitment and together they have a robust patient population sufficient to support this project (Magee Rehabilitation Hospital, Moss Spinal Cord Injury Center, and the Regional SCI Center of the Delaware Valley). Dr. Hiremath has had ongoing collaborations with the Co-Investigators in each site (Drs. Schmidt-Read, Ramos-Lamboy, Marino) and will work with each to optimize recruitment.
- The research and laboratory resources of the Principal Investigator and Co-Investigators are strong and support the computer, technology and other resources needed to implement this project.

Weaknesses

- None

Study Timeline:

Strengths

- Addresses each component of study

Weaknesses

- If subjects are enrolled in year 5 they will not finish 24 week FU until after the second quarter of year 5 (which only has 3 quarters). This may be very tight for data collection. Efforts to accelerate recruitment/enrollment is previous years, even slightly, might achieve more time for data analysis. With the complex analysis plan that might be an advantage.

Protections for Human Subjects:

Acceptable Risks and/or Adequate Protections

- This is anticipated to be a low-risk expedited study. Procedures for protection of HIPAA and confidentiality including use of smartphone are detailed and appropriate.

Data and Safety Monitoring Plan (Applicable for Clinical Trials Only):

Acceptable

- Plan is appropriate to this trial. Three individuals, Dr. Margaret A. Finley (Drexel University), Dr. Rochelle J. Mendonca (Columbia University), and a third scientist TBN will constitute the DSMB. The DSMB will review protocol prior to activation of the study and develop a data safety and monitoring plan. They also will review data annually to monitor adherence to the data safety plan and potential adverse events from study participation. Communication will be ongoing.

Inclusion Plans:

- Sex/Gender: Distribution justified scientifically
- Race/Ethnicity: Distribution justified scientifically
- For NIH-Defined Phase III trials, Plans for valid design and analysis: Scientifically acceptable
- Inclusion/Exclusion Based on Age: Distribution justified scientifically
- All plans are detailed and appropriate

Vertebrate Animals:

Not Applicable (No Vertebrate Animals)

Biohazards:

Acceptable

Resource Sharing Plans:

Acceptable

- Detailed and appropriate

Budget and Period of Support:

Recommend as Requested

THE FOLLOWING SECTIONS WERE PREPARED BY THE SCIENTIFIC REVIEW OFFICER TO SUMMARIZE THE OUTCOME OF DISCUSSIONS OF THE REVIEW COMMITTEE, OR REVIEWERS' WRITTEN CRITIQUES, ON THE FOLLOWING ISSUES:

PROTECTION OF HUMAN SUBJECTS: ACCEPTABLE

INCLUSION OF WOMEN PLAN: ACCEPTABLE

INCLUSION OF MINORITIES PLAN: ACCEPTABLE

INCLUSION ACROSS THE LIFESPAN: ACCEPTABLE

COMMITTEE BUDGET RECOMMENDATIONS: The budget was recommended as requested.

Footnotes for 1 R01 HD103904-01; PI Name: Hiremath, Shivayogi Vishwanath

NIH has modified its policy regarding the receipt of resubmissions (amended applications).See Guide Notice NOT-OD-18-197 at https://grants.nih.gov/grants/guide/notice-files/NOT-OD-18-197.html. The impact/priority score is calculated after discussion of an application by averaging the overall scores (1-9) given by all voting reviewers on the committee and multiplying by 10. The criterion scores are submitted prior to the meeting by the individual reviewers assigned to an application, and are not discussed specifically at the review meeting or calculated into the overall impact score. Some applications also receive a percentile ranking. For details on the review process, see http://grants.nih.gov/grants/peer_review_process.htm#scoring.
